# Editorial

**Published:** 1844-11-02

**Authors:** 


### THE MEDICAL EXAMINER.

PHILADELPHIA, NOV. 2, 1844.

### MEDICAL SCHOOLS OF PHILADELPHIA.

The regular courses of lectures in these institutions, commence on Monday next, the 4th inst. Of the size of the classes, it is yet impossible to speak—from present appearances, it is probable the number of students in attendance will be large. Already many are in the city, coming from all parts of the United States, South America, Nova Scotia, Canada, and even from Europe? We are pleased to observe that corresponding efforts are made to meet the high expectations which have led so many to this ancient and renowned seat of medical science, so that none shall go away disappointed. Extensive arrangements exist for clinical instruction, as well as didactic teaching, and every thing indicates a season of unwonted activity.

### NEW ORLEANS MEDICAL JOURNAL.

At last we have received the *second* number of our new contemporary, and find it well stored with rich and useful matter. We are apprised that it was regularly mailed at the time of publication, and of course our not receiving it sooner arose from some of the thousand accidents which lie in wait to disappoint Editors, in common with all others dependant on post office arrangements.

We find in the number before us a temperate and gentlemanly reply to our strictures on some remarks of our southern brethren, contained in their introductory address. We are glad to find that in some points we agree with them, although in the *peculiar opinion* which elicited our remarks, we do not agree; however, as the subject is ably handled in the communication of a correspondent, contained in the present number of the Examiner, we shall abstain, for the present at least, from any further discussion of the matter.

In reference to the statement of our correspondent, of what is or has been taught in the respectable western institution which he names, we know nothing. We had never heard, nor suspected, that the doctrine which he so well combats, was seriously promulgated by more than *one* individual: one who appears to think that a physician must acquire his education *exclusively* in the West or South, to be qualified to treat the diseases of those sections: that is, no matter how much he may know, or how much he may have learned in the South or West, if he has learned any thing of medicine in the North or the East, he is, ipso facto, disqualified for practising physic "in the valley of the Mississippi!" Our brethren in New Orleans, we are pleased to find, advocate no such absurdity.

### MEETING OF MEDICAL SUPERINTENDENTS OF HOSPITALS FOR THE INSANE.

One of the most interesting conventions that has been held in this city for a long time, both as it regards the objects for which it was held, and the individuals who composed it, took place week before last. A member of the body has politely furnished us with the following note of its proceedings, which we have much pleasure in laying before our readers. The movement is an important one, and we trust and believe that much good will result from it.

A meeting of the Medical Superintendents of Hospitals for the Insane, assembled at Jones' Hotel in the city of Philadelphia on the 16th of October, 1844, and continued its Sessions until the evening of the 20th.

Dr. Samuel B. Woodward of the Massachusetts State Lunatic Hospital was appointed President, Dr. S. White of the Hudson Lunatic Asylum, Vice President, and Dr. J. S. Kirkbride of the Pennsylvania Hospital for the Insane, Secretary and Treasurer.

The discussions of this body were of a highly interesting character, and were participated in by the following gentlemen representing most of the prominent institutions for the insane in the United States, viz.: Dr. Ray of the Maine Insane Hospital, Dr. Bell of the McLean Asylum, Dr. Woodward of the Mass. State Hospital, Dr. Stedman of the Boston Lunatic Hospital, Dr. Cutter of the Pepperell Institution, Dr. Butler of the Connecticut Retreat, Dr. Brigham of the N. York State Hospital, Dr. Earle of the Bloomingdale Asylum, Dr. White of the Hudson Lunatic Asylum, Dr. Kirkbride of the Pennsylvania Hospital for the Insane, Dr. Awl of the Ohio Lunatic Asylum, Dr. Stribling of the Western Asylum of Va., and Dr. Galt of the Eastern Asylum of Virginia.

Among the important matters referred to committees to report upon at a future meeting, were the medical and moral treatment of insanity—the construction and organization of Insane Hospitals—the Jurisprudence of Insanity—on restraint and restraining apparatus—on the Statistics of Insanity—on the support of the Pauper Insane, and on the best provision for insane prisoners. Before adjourning, the meeting adopted the title of "The Association of Medical Superintendents of American Institutions for the Insane," and elected as members, the medical superintendents of the various incorporated or other legally constituted institutions for the insane, now existing in the United States, or which may be commenced prior to the next meeting.

The Association adjourned to meet in the city of Washington, on the 2d Monday of May, 1846.

### SYDENHAM SOCIETY.

For the information of the members of this society residing in the United States, we extract the following advertisement from the cover of the last number, (October, 1844) of the "British and Foreign Medical Review." Professor Dunglison is authorized to receive subscriptions, and transmit the names of such as desire to become members.

"The Members of this Society are respectfully informed that the Council are about to make up the List of Members for the current year, and are anxious that all subscriptions should be paid immediately, in order that the list may be as complete as possible, and that the Council may be able to judge what funds will be at their disposal for this year. The number of members last year amounted to 1750. The three works issued were 'Hecker's Epidemics,' 'Louis on Phthisis,' and 'Sydenham's Works in

Latin.' The Council regret having been compelled to refuse many subscriptions for the first year, owing to the whole impression of the several works having been exhausted ; but as it was not deemed right to print many more copies than were required to supply the members registered at the time, the disappointment has been unavoidable. Nor will it be possible to prevent the recurrence of similar disappointments, unless the subscriptions are paid early.

Any member who may not have received the Second Report, or the whole of his books for the past year, may have them immediately on applying at the Office, and stating what books he has not received. Subscriptions may be remitted by post-office order, payable to the Treasurer or the Secretary, at the chief office, or through any London Banker. Any number of Reports for distribution by the members among their friends may be had on application at the Office of the Society, 45 Frith street, Soho.

By order of the Council,

JAS. RISDON BENNETT, M. D., Sec.

London, Sept. 12, 1844.

### MAL-PRACTICE.

*Trial of John Garland, Surgeon, accused of feloniously killing Mary Dent.*

The following account, which we extract from the London Lancet of the 24th of August last, is one of the most extraordinary, "take it all in all," in the annals of Surgery. The statement is from Mr. Mitchell, of Addenbrook's Hospital, Cambridge, one of the Surgeons who performed the post-mortem examination.

*Mr. Henry Mitchell's History of the Case.*

The name of the unfortunate patient was Mary Dent, wife of John Dent, of Littleport, in the Isle of Ely, labourer; she was twenty-three years of age, of good health, and robust appearance; she had borne five children, and had miscarried once; she was married at a very early age, and became a mother when between sixteen and seventeen years old.

On the 22d day of May last, Mary Dent complained of feeling very unwell; she felt great pain, and vomited occasionally, and being apprehensive of miscarriage, sent off, about eleven o'clock at night, for Mr. John Garland, a person of middle age, who has practised as a surgeon and accoucheur, at Littleport, ever since 1816.

It appears that on the day in question the patient had occasion to lift or drag a sack of flour, containing fourteen stone ; it also appears that, according to her own account, she was at this time about three months advanced in pregnancy, having menstruated for the last time on the 14th of February.

Upon his arrival, Mr. Garland proceeded to examine the patient, by passing his hand and arm into the vagina, intending, as he himself expressed it, to "bring the child." He shortly afterwards made a second examination, whereupon the patient intreated of him to desist, "for he was pulling her entrails out;" and presently Ann Banyard, the nurse, saw hanging out in the bed "a large quantity of entrails, as many as could lie on a large plate." (I quote her own words, as taken down by the coroner.)

When matters had arrived at this crisis, Mr. Garland appeared most anxious for further advice and assistance, and at his urgent request, a messenger was despatched to Ely, who returned, bringing with him Mr. Jones, of Ely, surgeon.

Upon turning down the bed-clothes, Mr. Jones discovered in the bed a something, which he at first mistook for the umbilical cord, but a more careful examination convinced him that the protruding mass was small intestine, depending from the vagina. Upon a minute inspection, he ascertained that the intestine was completely detached from the mesentery, throughout its whole length, and that it was extensively lacerated ; the distal portion being torn completely across, that is, its whole diameter being completely divided, whilst the proximal portion was lacerated, so as to be very nearly divided. Under these unfortunate and perplexing circumstances, Mr. Jones was of opinion that any attempt to save the intestine would prove useless, he therefore passed a ligature around the intestine, above the proximal laceration, and close to the vagina, and cut off all the intestine below the ligature ; the intestine, so cut off, measured nineteen feet six inches. Mr. Jones then took his departure, stating his belief that the patient could not survive many hours.

All this occurred between four and seven o'clock, on the morning of the 23d of May. Shortly after the departure of Messrs. Jones and Garland, the fœtus was found in the bed ; it appeared a fœtus of about three months. About twelve hours after the application of the ligature, the bowel above the ligature became very much distended, and ultimately burst. Subsequently the nurse, Ann Banyard, removed about half-a-yard more of intestine, without any medical man being present; she cut it off, and she did so "because it became very black and was very offensive."

The poor woman lingered seventeen days, and expired suddenly and at once, whilst attempting to raise herself up in bed, on the 8th day of June. During this period she did not suffer so acutely as might have been imagined ; for three or four days the stomach rejected everything, but latterly it became much less irritable ; her skin was cool, her pulse rarely above eighty, her countenance natural, and she complained of no pain, neither was there any appearance of hæmorrhage. She took a little simple sesqui-carbonate of soda as medicine, and weak gruel or chicken-broth as diet.

I was instructed to make a post-mortem examination, about forty eight hours after death. The following is the record of the examination :—

A very marked flatness and depression was observable between the two ilia ; over the whole abdomen the bodies of the vertebræ could be more distinctly felt than naturally they should be; the external parts of generation and the perinæum were very much excoriated. There was nothing else worthy of note about the trunk.

Upon opening the abdomen, the liver was ascertained to be healthy, as also the stomach. The omentum was offensive, black, gangrenous, and adherent to the arch of the colon and to the small intestines generally ; this adhesion was more marked from the symphysis-pubis to the right than to the left iliac region.

The colon appeared shrunken and contracted, and was so adherent to the omentum throughout the extent of the ascending and transverse portions, that the omentum and colon might be turned back together ; the whole of the ascending portion of the colon was in a state of complete gangrene ; over the region of the cæcum a small and circumscribed collection of matter was found, and it appeared as if, in this situation, the small intestines had been separated from the large ; the descending portion of the colon and the rectum were not in so diseased a condition.

About *two yards* only of small intestines remained in the abdomen, these, towards the lower portion, were very gangrenous, and upon tracing them downwards it was discovered that they were very adherent in the right iliac region, and that in this situation they dipped downwards and inwards to the right of the uterus, and became attached, by their lower margin, to the borders of an opening found in the right side of the vagina; the small intestines appeared to terminate in the vagina, for they could not be traced onwards to the large.

The mesentery was gangrenous, and had been torn in two or three places.

In the vagina was found, in the upper part and on the right side, a laceration sufficiently large to admit two or three of my fingers; this laceration was found to communicate with, and to lead into, the above-mentioned depending portion of small intestine, so that the fingers could readily be passed from t e vagina into the small intestine; the vagina on the left side was healthy and unruptured.

The uterus was normal in size and appearance, and what perhaps is rather singular, did not exhibit any traces of recent impregnation.

The bladder was healthy, so were the lungs, and so was the heart.

The jury found Mr. Garland *guilty*, and he was sentenced to one month's imprisonment.

After reading this statement, it is difficult to say which excites our astonishment most, the ignorance and brutality of Garland, which led him to introduce "his hand and arm into the vagina," in a mere case of threatened abortion, and drag forth nineteen feet of intestine,—the incautiousness of Mr. Jones in amputating it without counsel and assistance, and all, as we are told in a letter from him on the subject, to avoid exposing such a vagabond,—the wonderful tenacity of life of the poor creature which enabled her to survive such terrible violence *seventeen days*,—or the disgracefully inadequate punishment inflicted on the culprit by the Court!

